# PCB Sulfates in Serum from Mothers and Children in Urban and Rural U.S. Communities

**DOI:** 10.1021/acs.est.2c00223

**Published:** 2022-05-02

**Authors:** Duo Zhang, Panithi Saktrakulkla, Rachel F. Marek, Hans-Joachim Lehmler, Kai Wang, Peter S. Thorne, Keri C. Hornbuckle, Michael W. Duffel

**Affiliations:** † Interdisciplinary Graduate Program in Human Toxicology, The University of Iowa, Iowa City, Iowa 52242 United States; ¶ Department of Pharmaceutical Sciences & Experimental Therapeutics, The University of Iowa, Iowa City, Iowa 52242, United States; § Department of Civil and Environmental Engineering, The University of Iowa, Iowa City, Iowa 52242 United States; # IIHR-Hydroscience & Engineering, The University of Iowa, Iowa City, Iowa 52242, United States; □ Department of Occupational and Environmental Health, The University of Iowa, Iowa City, Iowa 52242, United States; ⊥ Department of Biostatistics, The University of Iowa, Iowa City, Iowa 52242 United States

**Keywords:** PCBs, organic pollutants, PCB sulfates, PCB metabolites, human serum

## Abstract

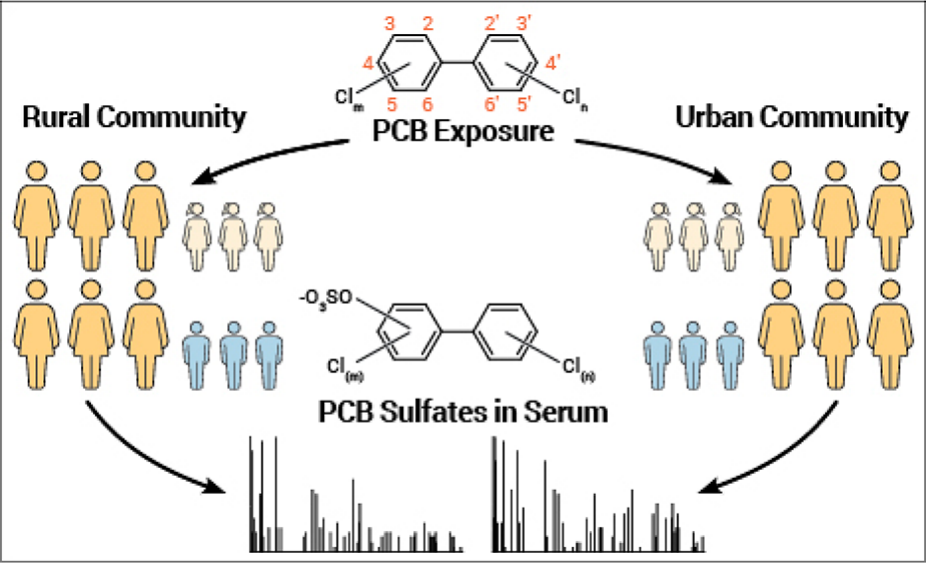

Serum samples from 24 subjects (6 mother–daughter and 6 mother–son dyads) in a rural community (Columbus Junction, Iowa) and 24 subjects (6 mother–daughter and 6 mother–son dyads) in an urban community (East Chicago, Indiana) were analyzed for 74 sulfated metabolites of polychlorinated biphenyls (PCBs). We detected significantly higher mean concentrations of total assessed PCB sulfates in the urban group (110–8900 ng/g fresh weight of serum, mean = 3400 ng/g, standard error = 300) than in the rural cohort (530–6700 ng/g fresh weight of serum, mean = 1800 ng/g, standard error = 500). Eight PCB sulfate congeners (4-PCB 2 sulfate, 4′-PCB 2 sulfate, 2′-PCB 3 sulfate, 4′-PCB 3 sulfate, 4-PCB 11 sulfate, 4′-PCB 18 sulfate, 4′-PCB 25 sulfate, and 4-PCB 52 sulfate) contributed over 90% of the total assessed PCB sulfates in most individuals. The serum samples were enriched in PCB sulfates with fewer than 5 chlorine atoms, and this congener distribution differed from those of PCBs and hydroxylated PCBs in previous studies in the same communities. Regression analysis indicated several significant congener-specific correlations in mother–child dyads, and these relationships differed by location and by mother–daughter or mother–son dyads. This is the first study reporting a broad range of PCB sulfates in populations from urban and rural areas.

## Abstract 2

This is the first study to quantify and compare multiple sulfated metabolites of environmentally and toxicologically relevant PCBs in individual serum samples from mothers and their children.

## Introduction

Polychlorinated biphenyls (PCBs) constitute a class of environmental contaminants that were originally produced and used as mixtures for many varied purposes until their intentional production was banned in the 1970s.^1,2^ These chemicals remain a global concern due to their environmental persistence from legacy production of PCBs. Additionally, their emerging presence as inadvertent contaminants resulting from the current manufacture of pigments, varnishes, and other products requires continued investigation of their impacts on human health and the environment.^3−6^ Many in vivo and in vitro studies have shown that polychlorinated biphenyls (PCBs) or their metabolites are responsible for a wide variety of toxic effects that include carcinogenesis, neurotoxicity, developmental toxicities, endocrine disruption, and others.^7−18^

Most studies on exposures and health effects in humans, however, have been focused on the concentrations of PCBs and their phenolic metabolites (OH-PCBs) in serum. For example, the AESOP (Airborne Exposure to Semivolatile Organic Pollutants) Study has previously incorporated analysis of the concentration of PCBs in air and food along with serum concentrations of PCBs and OH-PCBs in mothers and their children in Columbus Junction, Iowa (a rural community) and East Chicago, Indiana (an urban community).^19−22^ The urban community within East Chicago is highly industrialized and has a legacy of steel manufacturing, petroleum refining, and lead smelting; therefore, it is known to have relatively high exposures to PCBs, polycyclic aromatic hydrocarbons, and heavy metals.^21^ This community also has a known exposure source of PCBs from the Indiana Harbor and Ship Canal (IHSC), which flows within 0.5 km of the junior and senior high schools and from which ∼7.5 kg of PCBs evaporate per year.^23^ On the contrary, the Columbus Junction community in Iowa is a rural location without a known history of PCB sources derived from industrial manufacturing.^21^

Previous results from the AESOP Study indicated that East Chicago and Columbus Junction residents had similar concentrations of total PCBs (209 congeners analyzed, from nondetected to 658 ng/g lipid weight, median = 33.5 ng/g lw) and OH-PCBs (4 congeners analyzed, from nondetected to 0.07 ng/g lw) in serum samples collected between 2008 and 2009.^21^ This study also found a strong positive relationship between the sum of the detected OH-PCBs and the sum of their parent PCBs in serum.^21^ Furthermore, indoor air PCB concentrations were found to be the main factor affecting blood levels of PCBs via inhalation.^21,24,25^ This was also indicated by later research modeling dietary intake and inhalation exposures with detected serum PCB and OH-PCB levels, where PCB inhalation exposures (especially for lower-chlorinated volatile congeners) for some children were greater than their dietary exposure.^26^ Other AESOP Study results have shown that PCBs in meat and dairy products were the major sources of dietary exposure to PCBs in these two study populations, and there were strong associations among the identified PCB congener distributions and those PCBs present in Aroclors and in nonlegacy sources.^27^ Another component of the AESOP Study analyzed 58 OH-PCB congeners in serum samples collected between 2010 and 2011 and found total serum OH-PCBs in adolescents that ranged from 0.017 to 0.160 ng/g fresh weight (median 0.037 ng/g f.w.) and in mothers that ranged from 0.020 to 0.314 ng/g f.w. (median 0.063 ng/g f.w.).^22^ The main observations were that higher-chlorinated OH-PCBs (i.e., 5 or more chlorine atoms) predominated, and lower-chlorinated OH-PCB congeners were rarely observed in serum samples.^22^

The reasons for the observations of similar total serum concentrations of PCBs and OH-PCBs between the two communities that had different levels of background exposure to PCBs, however, have remained unresolved. One potential explanation for this finding is that conversion of the PCBs to metabolites differed between the two communities. Thus, with the first detection of PCB 11 sulfate in human serum from AESOP Study cohorts, we hypothesized that serum levels of some PCBs and OH-PCBs may not fully reflect actual PCB exposures in a human population due to sulfation of the OH-PCB metabolites and that the overall exposures to PCBs may be underestimated by only examining PCBs and OH-PCBs in serum.^28^ The sulfation of OH-PCBs is catalyzed by human cytosolic sulfotransferases and, thus, is part of a significant pathway for metabolism of PCBs.^18,29,30^ Our recent development of a procedure for analysis of multiple PCB sulfates in human serum has provided the methodology to investigate the prevalence of these sulfated PCB metabolites in the two populations that have been the subject of the AESOP Study.^31^ We have now applied this method for extraction and quantification of PCB sulfates in human serum to samples collected during 2015–2016 from 48 individuals living in urban and rural communities within the AESOP Study. Our results provide new insights into the potential reasons for unresolved questions relating to exposures and serum concentrations of PCBs and OH-PCBs.

## Materials and Methods

### Materials

Serum samples analyzed in this study were collected by AESOP Study personnel between December 2015 and May 2016 under University of Iowa Institutional Review Board-approved protocols (IRB 200604723) and stored before use as previously described.^19^ Serum samples from 48 study subjects were analyzed. A total of 24 individuals (6 mother–daughter pairs and 6 mother–son pairs) were from the Columbus Community School District, and 24 individuals (6 mother–daughter pairs and 6 mother–son pairs) were from East Chicago. The ages (median, (Q1, Q3)) of mothers and children at the time when serum samples were collected from Columbus Junction were 41.5 years, (34.5, 44.5) and 15.0 years, (14.0, 16.5), while those from East Chicago were 41.0 years, (38.5, 46.0) and 18.5 years, (17.0, 20.5). Blood samples were collected by bilingual AESOP Study staff at subjects’ homes and followed a standard venipuncture procedure.^21,22^ Blood was drawn into empty glass Vacutainer tubes and allowed to clot for 30 min before they were centrifuged to fully separate the cells from serum. The supernatant sera were transferred to glass vials with Teflon caps and stored at −20 °C until utilization as described previously.^19^

The following materials were purchased from Sigma-Aldrich (St. Louis, MO): *Helix pomatia* arylsulfatase (Type H-2, ≥2000 units/mL), NHS-Activated Sepharose 4 Fast Flow (GE17-096-01), l-tyrosine ethyl ester hydrochloride, sodium metavanadate (anhydrous, 99.9%), Millipore Centriprep centrifugal filter units (10K MWCO, cellulose), *p*-nitrophenyl sulfate, *p*-nitrophenyl glucuronide. Tris-HCl (Ultrapure, Molecular Biology grade) and Mops (Molecular Biology grade) were from RPI (Mt. Prospect, IL). All other reagents for enzyme purification and assay were from commercial sources and were ACS reagent grade or higher. The reagent water for aqueous solutions was Optima quality (ThermoFisher Scientific, Fair Lawn, NJ). Diazomethane was prepared by the Synthesis Core of the Iowa Superfund Research Program (ISRP) as previously described.^31^

The ^13^C-labeled OH-PCB mixture used as surrogate standard for OH-PCB analysis (containing 3′,4′-dichloro-4-[^13^C_12_]biphenylol, 2′,4′,5′-trichloro-4-[^13^C_12_]biphenylol, 2′,3′,4′,5′-tetrachloro-4-[^13^C_12_]biphenylol, 2′,3,4′,5,5′-pentachloro-4-[^13^C_12_]biphenylol, 2′,3,3′,4′,5,5′-hexachloro-4-[^13^C_12_]biphenylol, 2,2′,3,3′,4′,5,5′-heptachloro-4-[^13^C_12_]biphenylol, and 2,2′,3,4′,5,5′,6-heptachloro-4-[^13^C_12_]biphenylol) was purchased from Wellington Laboratories (Guelph, ON, Canada; product code MHPCB-MXA). PCB 204 (2,2′,3,4,4′,5,6,6′-octachlorobiphenyl) was obtained from AccuStandard (New Haven, CT) and deuterated-PCB 30 (2,4,6-trichlorinatedbiphenyl-d5) was from Cambridge Isotope Laboratories (Tewksbury, MA). As seen in Table S1, methoxy-PCB standards for GC-MS/MS quantitation were either prepared by the ISRP Synthesis Core or purchased from Wellington Laboratories (Guelph, ON), AccuStandard (New Haven, CT), CDN Isotopes (Pointe-Claire, QC) and Cambridge Isotope Laboratories (Tewksbury, MA), as indicated previously.^31^ All solvents for extraction and analysis of OH-PCBs were of pesticide residue analysis grade.

### Extraction of PCB Sulfates from Serum and Sulfatase-Catalyzed Hydrolysis to OH-PCBs

Serum collected from each individual originally weighed 4 g (approximately 4 mL). Samples were thawed at room temperature, weighed, and split into two equal parts (approximately 2 g each) in PYREX glass tubes (maximum volume of 15 mL) with rubber-lined phenolic caps. A surrogate standards mixture with a final concentration of 50 ng/mL was prepared in methanol. This mixture included ^13^C-4′-OH-PCB 12, ^13^C-4′-OH-PCB 29, ^13^C-4′-OH-PCB 61, ^13^C-4′-OH-PCB 120, ^13^C-4′-OH-PCB 159, ^13^C-4′-OH-PCB 172, and ^13^C-4′-OH-PCB 187. A total volume of 100 μL of surrogate standards (50 ng/mL) was spiked into each 2 g of serum sample. A 2 mL aliquot of 1% v/v formic acid was added to each sample and thoroughly mixed for 20 s, followed by addition of 6 mL of acetonitrile. Samples were fully mixed again and incubated at 4 °C for 2 h. Each sample was then centrifuged for 30 min at 3000*g*, and the supernatant layer was transferred into a new test tube containing approximately 100 mg of solid NaCl and approximately 300 mg of solid MgSO_4._ Supernatants with salts were then vortexed for at least 20 s, followed by 30 min centrifugation at 3000*g*. The organic layer of each sample was, then, transferred to a new test tube and gently evaporated under nitrogen flow to a final volume of approximately 0.5 mL. Approximately 1.5 mL of 200 mM sodium acetate buffer, pH 6.8, was added to each concentrated sample and vortexed. A total of 12.6 enzyme units (20 μL) of purified *Helix pomatia* sulfatase was added to half of the samples, while the other half of the samples received an equivalent volume of 200 mM sodium acetate buffer, pH 6.8. Preparation of the purified sulfatase was described previously,^31^ and it included purification by affinity chromatography using a modified procedure developed from Skorey et al.^32^ and verification of the removal of contaminating glucuronidase activity by assay with *p*-nitrophenyl glucuronide and *p*-nitrophenyl sulfate.^31^ Samples were then sealed with parafilm and incubated in a shaking water bath at 37 °C for 1 h.

### Extraction and Derivatization of OH-PCBs for GC-MS/MS Analysis

The extraction, separation, derivatization, and cleanup methods for OH-PCB quantification were carried out as described previously.^31^ Briefly, samples after incubation with and without purified sulfatase were denatured with 6 N hydrochloric acid (HCl) and 2-propanol, and this was followed by extraction with 1:1 (v/v) hexane: methyl *tert*-butyl ether (MTBE). The extracts were washed with 1% (w/w) potassium chloride (KCl) and, then, subjected to liquid–liquid partitioning with potassium hydroxide and hexane. The neutralized portion was discarded, and the OH-PCBs in the alkaline layer were reprotonated with 2 M HCl and extracted using 9:1 (v/v) hexane: MTBE. Samples containing OH-PCBs were, then, derivatized to the related methyl ethers (MeO-PCBs) using diazomethane as previously described.^31^ Lipid residues were removed by mixing with concentrated sulfuric acid, followed by sulfuric acid-activated silica gel column-washing with dichloromethane.^31^

### Quantification and Analysis of PCB Sulfates

After lipid removal and solvent exchange to hexane, sample extracts were concentrated to about 0.5 mL and transferred to standard 2.0 mL glass autosampler vials and spiked with 100 μL of internal standards (IS) mixture that contained PCB 204 (2,2′,3,4,4′,5,6,6′-octachlorobiphenyl) and deuterated-PCB 30 (2,4,6-trichlorinatedbiphenyl-*d*_5_), where each was present at a concentration of 100 ng/mL in hexane.^31^ Samples containing MeO-PCBs were either immediately analyzed by GC-MS/MS or stored at −10 °C prior to analysis. Prepared samples were analyzed with gas chromatography-triple quadrupole mass spectrometry (GC-MS/MS; Agilent 7890B GC and 7000D QqQ, Agilent Technologies, Santa Clara, CA) equipped with a Supelco SPB-Octyl capillary column (poly(50% *n*-octyl/50% methyl siloxane), 30 m × 0.25 mm i.d., 0.25 μm film thickness).

The amount of each congener was calculated through the application of a relative response factor and correction according to the percent recovery of surrogate standards on a per-sample basis. ^13^C-4′-OH-PCB 29 was used for mono- to tetra- chlorinated congeners, ^13^C-4′-OH-PCB 61 was used for penta-chlorinated congeners, ^13^C-4′-OH-PCB 159 was used for hexa-chlorinated congeners, ^13^C-4′-OH-PCB 187 was used for hepta-chlorinated congeners, and ^13^C-4′-OH-PCB 172 was used for octa-chlorinated congeners. The corrected concentrations of PCB sulfates obtained from serum were calculated using the recovery value of 42.9% as described previously.^31^ Conversion of the observed MeO-PCB (the end-products used for GC analysis) concentrations to the equivalent amount of parent PCB sulfates was performed based on the ratio of molecular mass values. The differences in concentrations of the MeO-PCBs from the sulfatase-added groups, and no-sulfatase-added groups were used for calculating initial PCB sulfate concentrations in serum samples based on sample weights. Concentrations were reported as picograms/gram of fresh weight serum instead of lipid weight (another common way of reporting serum PCB and OH-PCB levels) because of the higher hydrophilicity of PCB sulfates.

### Quality Control and Assessment

Sera from four individuals were analyzed per batch of samples (split to 8 samples for the purpose of parallel incubations), and 3 method blanks plus 1 reference standard were included along with each batch of serum samples. The reference standard tube contained only surrogate standards with the addition of 3 drops of methanol, and it was stored at −10 °C until the derivatization step. The MeO-PCBs quantified in the reference were assumed to yield the same percentage derivatization from spiked hydroxylated surrogate standards as in serum samples. Method blanks contained 1% (w/w) KCl in ddH_2_O as matrix. Method blanks went through the same experimental process as serum samples except for enzyme spiking. The surrogate standard recoveries in method blanks were the essential criteria for evaluating the reliability of manual operations as well as for determination of the congener specified limit of quantification (LOQ). Both Columbus Junction and East Chicago study groups had their own 18 method blanks for LOQ calculation. LOQs for the Columbus Junction and East Chicago groups are listed in Tables S2 and S3, respectively. Instrument blanks containing hexane were also analyzed before and after our calibration standards for each batch of study. Calibration range and linearity for the method were reported previously.^31^

### Statistical Analysis

Two sets of 95% quantile values of the 18 method blanks from each cohort were used as LOQ standards for selecting reported values of MeO-PCBs before calculating related PCB sulfate concentrations. Observations below LOQs were reported as 1/2 LOQs. Any calculated PCB sulfate congener concentration from a sulfatase-added sample that was equal to or less than its no-sulfatase-added parallel incubation was reported as zero. Note that the volume of each serum sample only allowed one measurement because of the previously determined limitations on the sensitivity of the method for PCB sulfates. Distribution of serum PCB sulfate concentrations in each of the two cohorts was checked by quantile–quantile plot to ensure normal distribution. For comparisons of significance between the two cohorts, Columbus Junction mothers versus East Chicago mothers and Columbus Junction children versus East Chicago children (including separated gender), *p* values were determined by two-sample student *t*-test assuming equal variance. Significance of the correlation coefficients between mother–daughter and mother–son dyads was assessed using simple linear regression. Profiles of detection frequencies for PCB congener groups in the two cohorts were compared by cos θ analysis as previously described.^20^

## Results and Discussion

**Figure 1:**
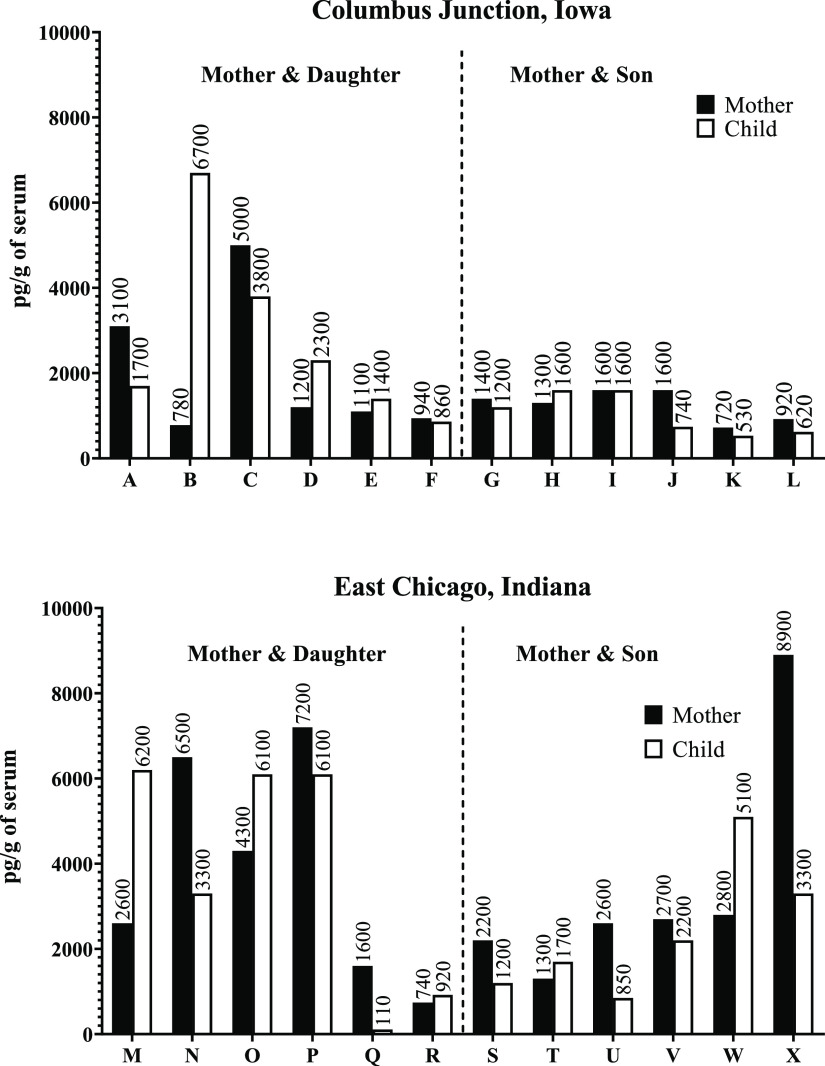
Total PCB sulfate concentrations in 48 individuals from the Columbus Junction and East Chicago cohorts. Alphabetical letters on the *x*-axis indicate individual mother–child dyads.

**Figure 2:**
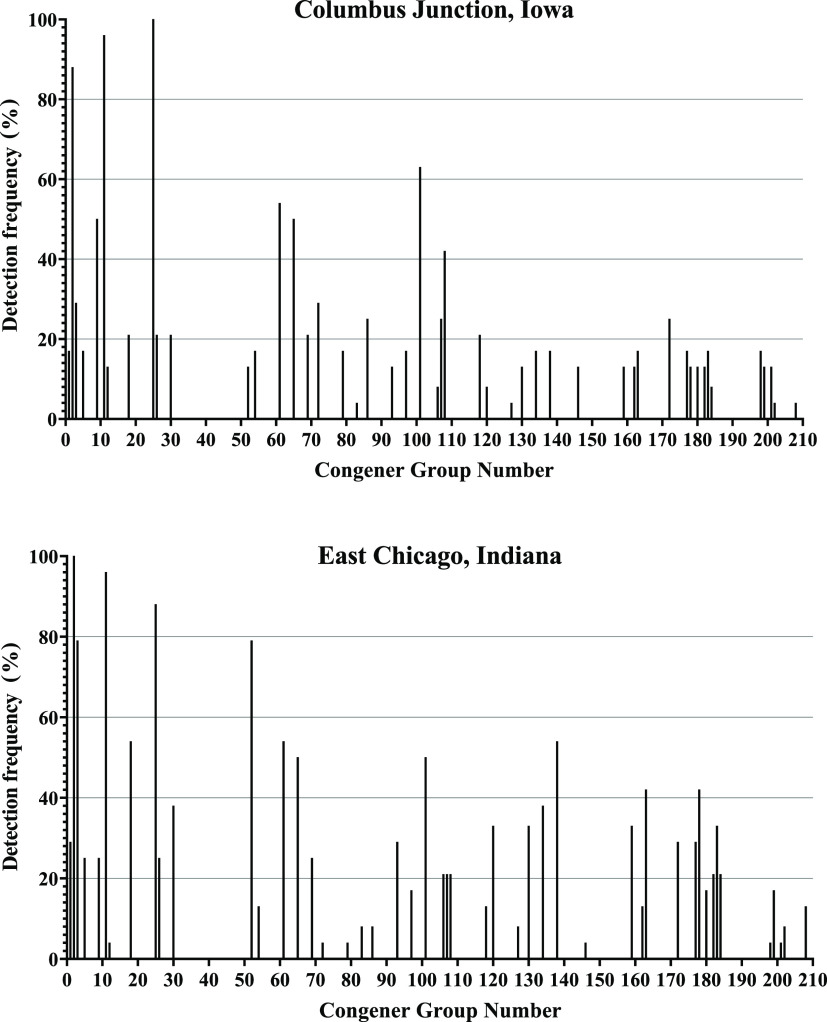
Detection frequencies of 48 PCB sulfate groups in 24 individuals from the Columbus Junction cohort and 24 individuals from the East Chicago cohort.

**Figure 3:**
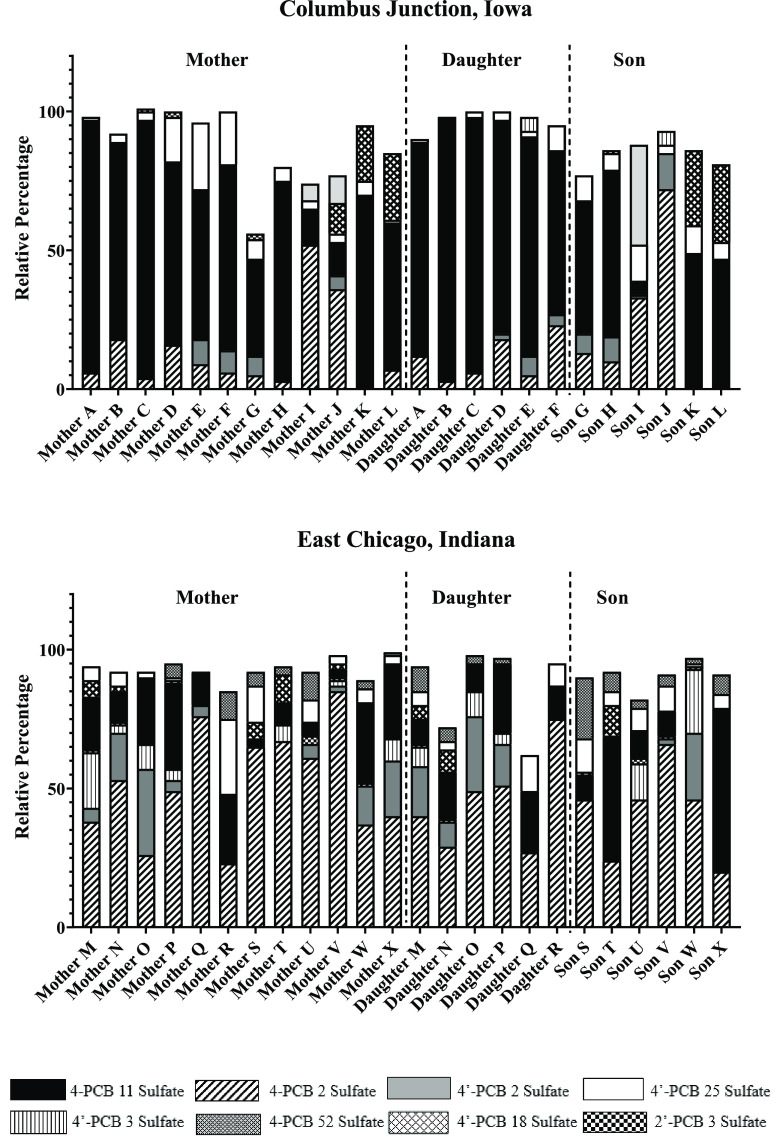
Percentages (%) of 8 major PCB sulfate congeners relative to total PCB sulfates in each of 48 individuals from the Columbus Junction and East Chicago cohorts. Mother–child dyads are indicated by the same alphabetic letter.

The total PCB sulfate concentrations (sum of 74 congeners) for each individual from the Columbus Junction cohort ranged from 530 to 6700 pg/g of serum with a mean of 1800 ng/g and a population standard error of 500 pg/g (*N* = 24). In the East Chicago cohort, the total PCB sulfate concentrations ranged from 110 to 8900 pg/g of serum with a mean of 3400 ng/g and population standard error of 300 pg/g (*N* = 24). The levels of total PCB sulfates in the East Chicago cohort were significantly higher than in the Columbus Junction cohort (*p* = 0.009) (Figure 1). Among these 74 PCB sulfate congeners, some could be categorized into groups based on the number and arrangement of chlorine atoms. The result of this categorization was a total of 48 PCB sulfate groups distributed from PCB 1 sulfate to PCB 208 sulfate (details listed in Table S4). The detection frequencies of these 48 PCB sulfate groups in both the Columbus Junction and East Chicago cohorts are shown in Figure 2, and the median and range of observed concentrations for each group is shown in Tables S5 and S6. The two cohorts had similar overall profiles of detection frequencies (Figure 2, cos θ = 0.87), although there were distinct differences with some congeners. Six PCB sulfate groups had ≥50% detection frequencies in both cohorts (Columbus Junction, East Chicago): PCB 2 sulfate (88%, 100%), PCB 11 sulfate (96%, 96%), PCB 25 sulfate (100%, 88%), PCB 61 sulfate (54%, 54%), PCB 65 sulfate (50%, 50%), and PCB 101 sulfate (63%,50%). One congener group (PCB 9 sulfate) in the Columbus Junction cohort had a 50% detection frequency, while this value was below 50% in the East Chicago group. There were four PCB sulfate groups with greater than 50% detection frequencies in the East Chicago cohort that exhibited below 50% detection frequency in the Columbus Junction cohort: PCB 3 sulfate (79%), PCB 18 sulfate (54%), PCB 52 sulfate (79%), and PCB 138 sulfate (54%). We observed 8 PCB sulfate congeners that contributed the most to the total PCB sulfates in these samples: 4-PCB 2 sulfate, 4′-PCB 2 sulfate, 2′-PCB 3 sulfate, 4′-PCB 3 sulfate, 4-PCB 11 sulfate, 4′-PCB 18 sulfate, 4′-PCB 25 sulfate, and 4-PCB 52 sulfate. The percentage (%) contributions of these 8 PCB sulfates to the total PCB sulfate concentrations are shown in Figure 3. As a percentage of the total PCB sulfates in individual serum samples, 4-PCB 11 sulfate was predominant in the Columbus Junction group compared to the East Chicago group, whereas PCB 2 sulfates comprised the major portion of PCB sulfate congeners seen in the East Chicago cohort.

**Figure 4:**
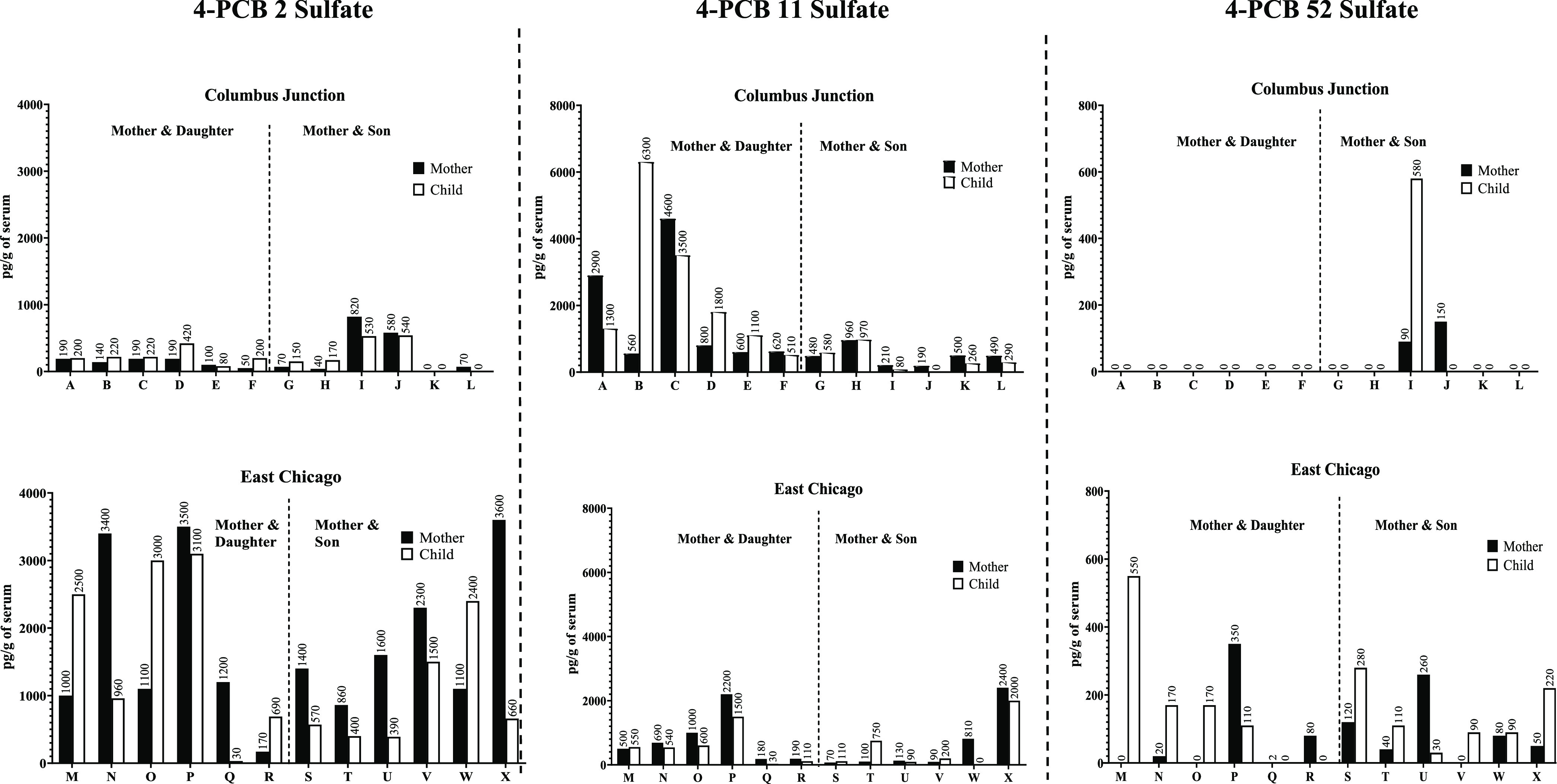
Comparisons of 4-PCB 2 sulfate, 4-PCB 11 sulfate, and 4-PCB 52 sulfate concentrations in 48 individuals from the Columbus Junction and East Chicago cohorts. Statistical *p* values between the two cohorts for each of these three compounds are 0.7 × 10^–4^ for 4-PCB 2 sulfate, 0.088 for 4-PCB 11 sulfate, and 0.029 for 4-PCB 52 sulfate.

Selected congener-specific comparisons of the serum PCB sulfate concentrations between individuals in the two cohorts are shown in Figures 4 and S1. 4-PCB 2 sulfate is a major contributor to the difference in monochlorinated PCB sulfates between the two populations (Figure 4). Additionally, 4′-PCB 2 sulfate, 2′-PCB 3 sulfate, and 4′-PCB 3 sulfate also contribute to this difference (Figure S1). It is particularly noteworthy that the sulfates derived from metabolism of PCB 11 and PCB 52 exhibit differences in their distributions in the sera of subjects from these two locations. Both PCBs are major constituents of indoor air samples in these communities,^19,20^ and both have been linked to neurotoxic responses.^9,10,33^ The extent to which differences in serum concentrations of the PCB sulfates might translate into neurotoxic or other responses, however, remains to be determined.

Correlation analyses of the 8 major PCB sulfates, total PCB 2 sulfate, and total PCB sulfates concentrations in mother–child dyads are summarized in Table S7. Three analytes, including total PCB 2 sulfates, 4-PCB 2 sulfate, and 4′-PCB 18 sulfate, showed correlations with significant *p* values in the Columbus Junction group. Three other analytes (2′-PCB 3 sulfate, 4-PCB 11 sulfate, and 4′-PCB 25 sulfate) exhibited correlations with significant *p* values in the East Chicago cohort. The correlation values for these 6 compounds were all 0.70 or above. A sex-separated regression analysis of the data is summarized in Table 1. No analyte showed significant correlation in mother-daughter pairs from the Columbus Junction group. Three analytes (total PCB 2 sulfates, 4-PCB 2 sulfate, and 4-PCB 11 sulfate) showed significant correlations in mother-son pairs from the Columbus Junction cohort. Three analytes (2′-PCB 3 sulfate, 4-PCB 11 sulfate, and 4′-PCB 18 sulfate) showed significant correlations in mother-daughter pairs from the East Chicago group, and one analyte (4-PCB 11 sulfate) exhibited significant correlation in mother-son pairs from the East Chicago group. It is also noteworthy that significant correlations between mother–daughter and mother–son dyads were not seen for several PCB sulfate congeners, and this may be reflective of differences either in exposure or in metabolism and disposition.

**Table 1:** Correlation Coefficients of PCB Sulfate Concentrations in Mother–Child Dyads (Sex-Separated)^a^

	Columbus Junction, IA		East Chicago, IN	
	mother–daughter	mother–son	mother–daughter	mother–son
total PCB sulfates	0.024 (0.96)	0.63 (0.18)	0.59 (0.22)	0.36 (0.48)
total PCB 2 sulfates	0.11 (0.83)	0.89 (0.017)	0.31 (0.56)	0.15 (0.78)
4-PCB 2 sulfate	0.52 (0.31)	0.93 (0.006)	0.26 (0.62)	0.13 (0.80)
4′-PCB 2 sulfate	0.79 (0.059)	0.45 (0.37)	0.52 (0.29)	0.0069 (0.10)
2′-PCB 3 sulfate	0.66 (0.16)	0.19 (0.72)	0.98 (0.0005)	0.48 (0.34)
4′-PCB 3 sulfate		0.21 (0.70)	0.85 (0.061)	0.27 (0.60)
4-PCB 11 sulfate	0.070 (0.90)	0.94 (0.005)	0.98 (0.0008)	0.84 (0.035)
4′-PCB 18 sulfate	0.030 (0.95)	0.71 (0.11)	0.93 (0.007)	0.65 (0.16)
4′-PCB 25 sulfate	0.50 (0.32)	0.29 (0.58)	0.32 (0.54)	0.16 (0.76)
4-PCB 52 sulfate	0.031 (0.94)	0.36 (0.49)	0.22 (0.67)	0.27 (0.61)

a Regression analyses were determined from 6 mother–daughter and 6 mother–son dyads from each of the two populations. Numbers are correlation coefficients (*p*-values). *p*-values were obtained from simple regression analysis. Significant correlation coefficients (i.e., *p* < 0.05) are in bold. Total PCB 2 Sulfates include 2-PCB 2 sulfate + 2′-PCB 2 sulfate + 6-PCB 2 sulfate + 5-PCB 2 sulfate + 3′-PCB 2 sulfate + 4-PCB 2 sulfate + 4′-PCB 2 sulfate.

While our findings on serum concentrations of PCB sulfates in these 48 individuals from urban and rural U.S communities expanded our current knowledge about PCB metabolites in humans, many questions remain. One initial question is what are the parent PCBs for these sulfated metabolites? Metabolism of PCBs can be complicated to predict since there are many possible pathways that depend upon congener structure, as well as factors as diverse as age, gender, diet, disease states, medication history, other xenobiotic exposures, tissue-dependent expression of xenobiotic-metabolizing enzymes, and others. One example of the complexity of pathways is the recent finding that there were 30 metabolites observed in HepG2 cells treated with PCB 11, and these included monohydroxylated, dihydroxylated, methoxylated-hydroxylated, and methoxylated-dihydroxylated derivatives, as well as corresponding sulfate and glucuronide conjugates.^34^ Another example is the identification of monohydroxylated, sulfate, and glucuronide metabolites in HepG2 cells exposed to PCB 3.^35^

To begin consideration of potential sources of the PCB sulfates observed in these serum samples, a preliminary retro-analysis of the 8 major PCB sulfate metabolites back to their possible precursor OH-PCBs and PCBs is shown in supplemental Figure S2. The main metabolic pathways of PCB sulfate formation include initial oxidation of PCBs to form related OH-PCBs catalyzed by hepatic cytochrome P-450 enzymes (CYPs), and this is followed by further transformation to PCB sulfates through reactions catalyzed by sulfotransferases.^18^ An important factor that must be considered during this process of formation of OH-PCBs from their parent PCBs is the potential for removal of an adjacent chlorine that may occur as a result of a 1,2-shift (NIH-shift) reaction after initial arene oxide formation.^36^ In vivo metabolic dechlorination of PCBs has been previously reported, and this may involve NIH-shift reactions, as well as mechanisms that do not require an arene oxide intermediate.^37,38^ Dechlorination of PCBs may also take place under anaerobic conditions, where the chlorine atoms in the meta and para positions can be removed more readily than those in the ortho position.^39^ Thus, when the metabolic sources of OH-PCBs and the potential for dechlorination reactions are considered, there are at least 10 possible PCBs that might serve as precursors to the 8 major PCB sulfates found in the serum samples of this study. These PCBs include PCB 2 (generates 4- and 4′-PCB 2 sulfates), PCB 3 (generates 2′- and 4′-PCB 3 sulfate, 4-PCB 2 sulfate), PCB 11 (generates 4-PCB 11 sulfate and sulfates generated from PCB 2), PCB 13 (generates 2′- and 4′-PCB 3 sulfate, and 4-PCB 11 sulfate), PCB 18 (generates 4′-PCB 18 sulfate), PCBs 25 and 28 (generate PCB 25 sulfate), and PCBs 49 and 52 (generate 4-PCB 52 sulfate). Previous studies have shown that PCB 28 is a precursor of 4-OH PCB 25 in human serum.^40^ Furthermore, PCB 28 is one of the most prevalent congeners detected in air samples.^18^ Among these potential precursor PCBs, PCB 11 is the only one not found in original Aroclor mixtures.^41^

An additional route for metabolic formation of PCB sulfates in humans could potentially involve direct exposure to the OH-PCBs that then serve as substrates for sulfation. OH-PCBs are present in the environment through both biotic and abiotic mechanisms.^42−46^ Moreover, in the case of one of the more prevalent PCB sulfates that we observed in serum, 4-PCB 2 sulfate, there is an industrial use of its precursor OH-PCB: 4-OH PCB 2 (also named as 2-chloro-4-phenylphenol) is a component of two commercial microbicides.^47^ The extent to which direct exposure to OH-PCBs might contribute to the PCB sulfates observed in human serum, however, remains to be determined.

**Figure 5:**
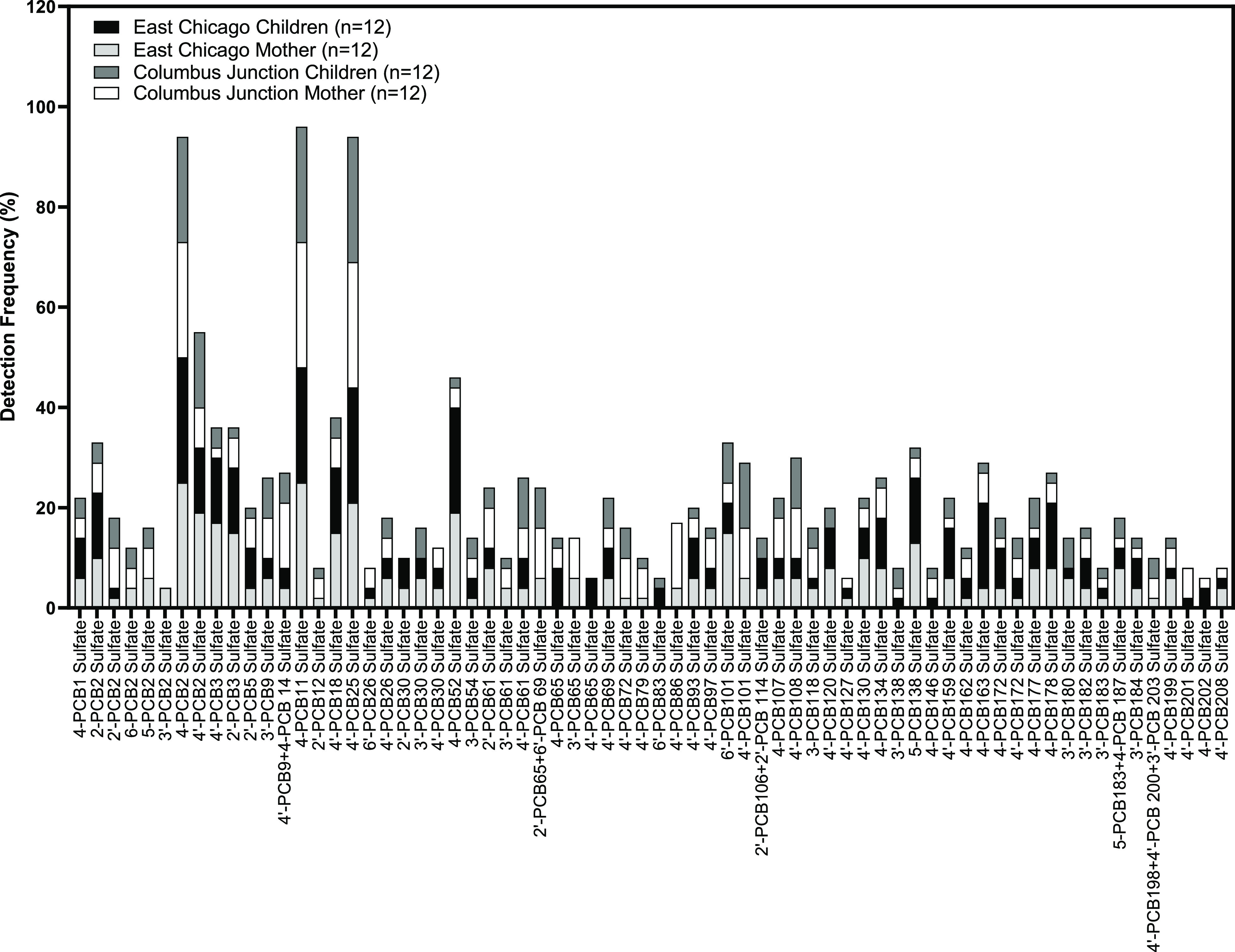
Detection frequency of each PCB sulfate congener in East Chicago and Columbus Junction children and their mothers.

It is particularly interesting that the trend of the overall distribution of PCB sulfate congeners seen in Figure 5 (i.e., the concentration of lower chlorinated vs higher chlorinated PCB sulfate congeners) was opposite to the profiles of lower chlorinated and higher chlorinated OH-PCB congeners detected in serum samples (Figure S3).^22^ It was not possible to perform a simultaneous determination of the congener distribution profile of PCBs or OH-PCBs in our samples due to limitations on the detection sensitivity for PCB sulfates, differences in sample processing methods, and the volume of serum that was collected from subjects in the study. We note, however, that a previous AESOP Study publication that compared PCBs in human sera collected from other subjects in the same Columbus Junction and East Chicago communities found similar levels of total PCB concentrations between these two populations, with only subtle differences observed in the concentrations of some individual PCB congeners.^21^ In contrast, we observed significantly higher levels of total PCB sulfates in the East Chicago group compared with the Columbus Junction group (*p* = 0.009), and differences in individual congeners were also seen. Previous determinations of the congener distribution of PCBs in serum samples from other subjects within these same two communities resembled the congener distribution of the OH-PCBs in serum more than they resembled the congener profiles of PCBs seen in air samples (i.e., enriched in higher chlorinated congeners).^19−22^ In our current study, the overall enrichment of lower chlorinated congeners of PCB sulfates was, however, more similar to the profiles of PCB congeners previously quantified in air samples that were collected in these same communities.^19,20^

Our observation of significant differences in total PCB sulfate levels between subjects in the Columbus Junction study population and the East Chicago cohort indicates that the PCB sulfates might be useful biomarkers for assessing some PCB exposures in human populations. A clear understanding of this possibility, however, will await a more complex study that specifically links exposures to PCBs in air and diet to serum concentration profiles of PCBs, OH-PCBs, and PCB sulfates that are simultaneously determined in the same individuals. Such simultaneous determination of PCBs, OH-PCBs, and PCB-sulfates would also be necessary to assess the usefulness of PCB sulfates as biomarkers for the route of exposure (e.g., inhalation).

It is also important to note that PCB sulfates themselves may contribute to various toxic responses due to their retention through binding to serum proteins,^48,49^ cellular uptake,^50,51^ and intracellular hydrolysis to more toxic OH-PCBs catalyzed by microsomal sulfatases.^52^ In the case of retention of OH-PCBs because of sulfation and subsequent hydrolysis, the conversion of some OH-PCBs into PCB quinone metabolites is recognized as a metabolic activation pathway in chemical carcinogenesis.^7,17,18^ It has also been shown that electrophilic biphenyl quinones can, depending upon the chemical structure, either increase or decrease the catalytic activity of a human sulfotransferase.^53^ Our current findings on the occurrence and distribution of PCB sulfate congeners in individual human subjects represents an initial step in understanding these complex relationships among exposures, metabolism, and toxicities for PCBs, OH-PCBs, and their sulfated metabolites.

## Supporting Information Available

Additional details on the sources of MeO-PCBs used as callibration standards, the LOQs for samples in the two cohorts, the definition of PCB sulfate congener groups used in the analyses, the frequency of detection and median concentrations of the PCB sulfates detected in the two study groups, a correlation analysis of the PCB sulfate concentrations in mothers and children from each of the two cohorts, a comparison of the concentrations of 4′-PCB 2 sulfate, 2′-PCB 3 sulfate, and 4′-PCB 3 sulfate in all individuals from each of the two study populations, a retro-analysis of the 8 most prevalent PCB sulfates showing potential precursor PCBs, and a previously published plot of the frequency of detection of OH-PCB congeners in the serum of other AESOP Study subjects in Columbus Junction, IA, and East Chicago, IN (PDF)

## Notes

The original data underlying this study are openly available as a data set in the Iowa Research Online repository at DOI: 10.25820/data.006162.
