# Supplementary material for: PCB Sulfates in Serum from Mothers and Children in Urban and Rural U.S. Communities
Source: Environ Sci Technol. 2022 May 2;56(10):6537–47. doi: 10.1021/acs.est.2c00223 (PMC9118556; doi:10.1021/acs.est.2c00223)
Supplement: Supplementary file 1 — es2c00223_si_001.pdf [file es2c00223_si_001.pdf]

SUPPORTING INFORMATION

PCB Sulfates in Serum from Mothers and Children in Urban and Rural U.S. Communities

Duo Zhang[1,2], Panithi Saktrakulkla[1,3], Rachel F. Marek[3,4], Hans-Joachim Lehmler[5], Kai Wang[6], Peter S. Thorne[5], Keri C. Hornbuckle[1,3,4], and Michael W. Duffel[1,2]*

[1] Interdisciplinary Graduate Program in Human Toxicology, [2] Department of Pharmaceutical Sciences & Experimental Therapeutics, [3] Department of Civil and Environmental Engineering, [4] IIHR-Hydroscience & Engineering, [5] Department of Occupational and Environmental Health, and [6] Department of Biostatistics, The University of Iowa, Iowa City, IA 52242

Additional data on individual congeners in each serum sample are available at: doi:10.25820/data.006162

Number of Pages: 18

Number of Figures: 3

Number of Tables: 7

Table of Contents

Table S1. Sources of MeO-PCBs used as calibration standards……………………………..S3

Table S2. LOQs (95% quantile of 18 method blanks) for Columbus Junction, IA, N=18.. S5

Table S3. LOQs (95% quantile of 18 method blanks) for East Chicago, I*N*, N=18 ....S6

Table S4. Composition of PCB sulfate congener groups S7

Table S5. Frequency of detection (DF), median and range (5th-95th) of the PCB sulfates detected in mothers and children from Columbus Junction...S9

Table S6. Frequency of detection (DF), median and range (5th-95th) of the PCB sulfates detected in mothers and children from East Chicago. S12

Table S7. Correlation analysis of PCB sulfate concentrations in mothers-children dyads..S15

Figure S1. Comparisons of 4'-PCB 2 sulfate, 2'-PCB 3 sulfate, and 4'-PCB 3 sulfate concentrations in 48 individuals from the Columbus Junction and East Chicago cohorts….S16

Figure S2. Preliminary proposed retro analysis of 8 major PCB sulfates to their parent PCBs. S17

Figure S3. Detection frequency of OH-PCB congeners in East Chicago and Columbus Junction adolescents and their mothers. *(*Koh, W.X., *et al. Chemosphere* **2016***,* 147, 389-395).........S18

Table S1. Sources of MeO-PCBs used as calibration standards

Name	Abbreviation	Source
**4-methoxy-2-chlorobiphenyl**	4-MeO-PCB 1	AccuStandard
**2-methoxy-3-chlorobiphenyl**	2-MeO-PCB 2	AccuStandard Custom
**2-methoxy-3'-chlorobiphenyl**	2'-MeO-PCB 2	AccuStandard Custom
**3-methoxy-3'-chlorobiphenyl**	3'-MeO-PCB 2	AccuStandard Custom
**4-methoxy-3-chlorobiphenyl**	4-MeO-PCB 2	AccuStandard
**4-methoxy-3'-chlorobiphenyl**	4'-MeO-PCB 2	AccuStandard Custom
**3-methoxy-5-chlorobiphenyl**	5-MeO-PCB 2	AccuStandard Custom
**2-methoxy-5-chlorobiphenyl**	6-MeO-PCB 2	AccuStandard
**2-methoxy-4'-chlorobiphenyl**	2’-MeO-PCB 3	ISRP Synthesis Core
**4-methoxy-4'-chlorobiphenyl**	4'-MeO-PCB 3	AccuStandard
**2-methoxy-2',3'-dichlorobiphenyl**	2'-MeO-PCB 5	AccuStandard
**3-methoxy-2',5'-dichlorobiphenyl**	3'-MeO-PCB 9	AccuStandard
**4-methoxy-2',5'-dichlorobiphenyl**	4'-MeO-PCB 9	AccuStandard
**4-methoxy-3, 3’-dichlorobiphenyl**	4-MeO-PCB 11	ISRP Synthesis Core
**2-methoxy-3',4'-dichlorobiphenyl**	2'-MeO-PCB 12	AccuStandard
**4-methoxy-3,5-dichlorobiphenyl**	4-MeO-PCB 14	AccuStandard
**4-methoxy-2,2',5'-trichlorobiphenyl**	4'-MeO-PCB 18	AccuStandard
**4-methoxy-2',3,4'-trichlorobiphenyl**	4’-MeO-PCB 25	ISRP Synthesis Core
**4-methoxy-2',3,5'-trichlorobiphenyl**	4'-MeO-PCB 26	AccuStandard
**2-methoxy-2',5,5'-trichlorobiphenyl**	6'-MeO-PCB 26	AccuStandard
**2-methoxy-2',4',6'-trichlorobiphenyl**	2'-MeO-PCB 30	AccuStandard
**3-methoxy-2',4',6'-trichlorobiphenyl**	3'-MeO-PCB 30	AccuStandard
**4-methoxy-2',4',6'-trichlorobiphenyl**	4'-MeO-PCB 30	AccuStandard
**3-methoxy-2,2',6,6'-tetrachlorobiphenyl**	3-MeO-PCB 54	AccuStandard
**2-methoxy-2',3',4',5'-tetrachlorobiphenyl**	2'-MeO-PCB 61	AccuStandard
**3-methoxy-2',3',4',5'-tetrachlorobiphenyl**	3'-MeO-PCB 61	AccuStandard
**2,3,4,5-tetrachloro-4'-methoxybiphenyl**	4'-MeO-PCB 61	Wellington Labs Mix A
**4-methoxy-2,2’,5,5’-tetrachlorobiphenyl**	4-MeO-PCB 52	ISRP Synthesis Core
**2-methoxy-2',3',5',6'-tetrachlorobiphenyl**	2'-MeO-PCB 65	AccuStandard
**3-methoxy-2',3',5',6'-tetrachlorobiphenyl**	3'-MeO-PCB 65	AccuStandard
**4-methoxy-2,3,5,6-tetrachlorobiphenyl**	4-MeO-PCB 65	AccuStandard Custom
**4-methoxy-2',3',5',6'-tetrachlorobiphenyl**	4'-MeO-PCB 65	AccuStandard
**4-methoxy-2',3',4',6'-tetrachlorobiphenyl**	4'-MeO-PCB 69	AccuStandard
**2-methoxy-2',4',5,6'-tetrachlorobiphenyl**	6'-MeO-PCB 69	AccuStandard
**4-methoxy-2',3,5,5'-tetrachlorobiphenyl**	4'-MeO-PCB 72	AccuStandard
**3,3',4',5-tetrachloro-4-methoxybiphenyl**	4'-MeO-PCB 79	Wellington Labs Mix B
**2-methoxy-2',3',5,5',6-pentachlorobiphenyl**	6'-MeO-PCB 83	AccuStandard
**4-methoxy-2,2',3',4',5'-pentachlorobiphenyl**	4'-MeO-PCB 86	AccuStandard
**4-methoxy-2,2',3',5',6'-pentachlorobiphenyl**	4'-MeO-PCB 93	AccuStandard
**2,2',3,4',5'-pentachloro-4-methoxybiphenyl**	4'-MeO-PCB 97	Wellington Labs Mix G
**2,2',4,5,5'-pentachloro-4'-methoxybiphenyl**	4'-MeO-PCB 101	Wellington Labs Mix B
**2-methoxy-2',3,4',5',6-pentachlorobiphenyl**	6'-MeO-PCB 101	AccuStandard
**2-methoxy-2',3',4',5,5'-pentachlorobiphenyl**	2'-MeO-PCB 106	AccuStandard
**2,3,3',4',5-pentachloro-4-methoxybiphenyl**	4-MeO-PCB 107	Wellington Labs Mix F

**2,3,3',4,5'-pentachloro-4'-methoxybiphenyl**	4'-MeO-PCB 108	Wellington Labs Mix E
**2,3,4,4',5-pentachloro-2'methoxybiphenyl**	2'-MeO-PCB 114	Wellington Labs Mix C
**2,3',4,4',5-pentachloro-3-methoxybiphenyl**	3-MeO-PCB 118	Wellington Labs Mix D
**2,3',4,5,5'-pentachloro-4'-methoxybiphenyl**	4'-MeO-PCB 120	Wellington Labs Mix A
**3,3',4,5,5'-pentachloro-4'-methoxybiphenyl**	4'-MeO-PCB 127	Wellington Labs Mix H
**2,2',3,3',4',5-hexachloro-4-methoxybiphenyl**	4'-MeO-PCB 130	Wellington Labs Mix E
**2,2',3,3',5,6-hexachloro-4-methoxybiphenyl**	4-MeO-PCB 134	Wellington Labs MiX B
**2,2',3',4,4',5-hexachloro-3-methoxybiphenyl**	3'-MeO-PCB 138	Wellington Labs Mix D
**5-methoxy-2,2',3,4,4',5'-hexachlorobiphenyl**	5-MeO-PCB 138	AccuStandard
**2,2',3,4',5,5'-hexachloro-4-methoxybiphenyl**	4-MeO-PCB 146	Wellington Labs Mix C
**2,3,3',4,5,5'-hexachloro-4'-methoxybiphenyl**	4'-MeO-PCB 159	Wellington Labs Mix G
**2,3,3',4',5,5'-hexachloro-4-methoxibiphenyl**	4-MeO-PCB 162	Wellington Labs Mix I
**2,3,3',4',5,6-hexachloro-4-methoxybiphenyl**	4-MeO-PCB 163	Wellington Labs Mix F
**2,2',3,3',4,5,5'-heptachloro-4'-methoxybiphenyl**	4'-MeO-PCB 172	Wellington Labs Mix H
**2,2',3,3',4',5,6-heptachloro-4-methoxybiphenyl**	4-MeO-PCB 177	Wellington Labs Mix F
**2,2',3,3',5,5',6-heptachloro-4-methoxybiphenyl**	4-MeO-PCB 178	Wellington Labs MiX B
**2,2',3,4,4',5,5'-heptachloro-3'-methoxybiphenyl**	3'-MeO-PCB 180	Wellington Labs Mix G
**2,2',3,4,4',5,6'-heptachloro-3'-methoxybiphenyl**	3'-MeO-PCB 182	Wellington Labs Mix C
**2,2',3',4,4',5,6'-heptachloro-3-methoxybiphenyl**	3'-MeO-PCB 183	Wellington Labs Mix D
**5-methoxy-2,2',3,4,4',5'6'-heptachlorobiphenyl**	5-MeO-PCB 183	AccuStandard
**2,2',3,4,4',6,6'-heptachloro-3'-methoxybiphenyl**	3'-MeO-PCB 184	Wellington Labs Mix A
**2,2',3,4',5,5',6-heptachloro-4-methoxybiphenyl**	4-MeO-PCB 187	Wellington Labs Mix E
**2,3,3',4',5,5',6-heptachloro-4-methoxybiphenyl**	4-MeO-PCB 193	Wellington Labs Mix I
**2,2',3,3',4,5,5',6-octachloro-4'-methoxybiphenyl**	4'-MeO-PCB 198	Wellington Labs Mix D
**2,2',3,3',4',5,5',6-octachloro-4-methoxybiphenyl**	4'-MeO-PCB 199	Wellington Labs Mix E
**2,2',3,3',4,5,6,6'-octachloro-4'-methoxybiphenyl**	4'-MeO-PCB 200	Wellington Labs Mix F
**2,2',3,3',4',5,6,6'-octachloro-4-methoxybiphenyl**	4'-MeO-PCB 201	Wellington Labs Mix B
**2,2',3,3',5,5',6,6'-octachloro-4-methoxybiphenyl**	4-MeO-PCB 202	Wellington Labs Mix A
**2,2',3,4,4',5,5',6-octachloro-3'-methoxybiphenyl**	3'-MeO-PCB 203	Wellington Labs Mix C
**2,2',3,3',4,5,5',6,6'-nonachloro-4'-methoxybiphenyl**	4'-MeO-PCB 208	Wellington Labs Mix C
**2,4,6-trichlorobiphenyl - 2',3',4',5',6'-d5**	d-PCB 30	Cambridge Isotope Labs
**2,2',3,4,4',5,6,6'-octachlorobiphenyl**	PCB 204	AccuStandard
**2,4,5-trichloro-4'-methoxy[13C]biphenyl**	13C 4'-MeO-PCB 29	Wellington Labs 13C
**2,3,4,5-4'-tetrachloro-4'-methoxy[13C]biphenyl**	13C 4'-MeO-PCB 61	Wellington Labs 13C
**2,3',4,5,5'-pentachloro-4'-methoxy[13C]biphenyl**	13C 4'-MeO-PCB 120	Wellington Labs 13C
**2,3,3',4,5,5'-hexachloro-4'-methoxy[13C]biphenyl**	13C 4'-MeO-PCB 159	Wellington Labs 13C
**2,2',3,3',4,5,5'-heptachloro-4'-methoxy[13C]biphenyl**	13C 4'-MeO-PCB 172	Wellington Labs 13C
**2,2',3,3',4,5,5'-heptachloro-4-methoxy[13C]biphenyl**	13C 4-MeO-PCB 187	Wellington Labs 13C

Table S2. LOQs (95% quantile of 18 method blanks) for Columbus Junction, IA, N=18

congener name	pg/mL of 1% KCl	congener name	pg/mL of 1% KCl
**2-MeO-PCB2**	4.82	**4'-MeO-PCB120**	10.17
**2'-MeO-PCB2**	2.41	**4'-MeO-PCB86**	4.91
**6-MeO-PCB2**	2.71	**4'-MeO-PCB97**	4.00
**4-MeO-PCB1**	3.04	**4'-MeO-PCB108**	2.09
**5-MeO-PCB2**	0.78	**3-MeO-PCB118**	5.06
**3'-MeO-PCB2**	2.86	**4-MeO-PCB107**	5.74
**4-MeO-PCB2**	37.42	**4'-MeO-PCB127**	3.79
**4'-MeO-PCB2**	24.02	**4-MeO-PCB134**	4.00
**4'-MeO-PCB3**	24.87	**4-MeO-PCB146**	3.67
**2'-MeO-PCB5**	2.97	**3'-MeO-PCB138**	2.85
**3'-MeO-PCB9**	1.20	**4'-MeO-PCB130**	5.54
**4'-MeO-PCB9+ 4-MeO-PCB14**	4.02	**4-MeO-PCB163**	1.89
		**5-MeO-PCB138**	5.11
**2'-MeO-PCB12**	6.35	**4'-MeO-PCB159**	3.83
**2'-MeO-PCB30**	3.23	**4-MeO-PCB162**	3.08
**3'-MeO-PCB30**	1.94	**3'-MeO-PCB184**	10.67
**6'-MeO-PCB26**	3.30	**4-MeO-PCB178**	3.93
**4'-MeO-PCB18**	39.45	**3'-MeO-PCB183**	3.12
**4'-MeO-PCB30**	10.89	**3'-MeO-PCB182**	7.53
**4'-MeO-PCB26**	8.33	**5-MeO-PCB183**	7.26
**3-MeO-PCB54**	3.21	**4-MeO-PCB187**	2.84
**2'-MeO-PCB65+ 6'-MeO-PCB69**	1.15	**4-MeO-PCB177**	2.86
		**3'-MeO-PCB180**	3.44
**4-MeO-PCB65**	3.00	**4'-MeO-PCB172**	3.35
**3'-MeO-PCB65**	7.09	**4'-MeO-PCB172**	1.87
**2'-MeO-PCB61**	18.14	**4-MeO-PCB202**	6.95
**4'-MeO-PCB72**	7.26	**4'-MeO-PCB201**	2.73
**4'-MeO-PCB69**	8.17	**4'-MeO-PCB198+ 4'-MeO-PCB200+ 3'-MeO-PCB203**	1.46
**4'-MeO-PCB65**	10.01		
**3'-MeO-PCB61**	4.86		
**4'-MeO-PCB61**	2.33	**4'-MeO-PCB199**	1.60
**4'-MeO-PCB79**	3.41	**4'-MeO-PCB208**	22.56
**6'-MeO-PCB101**	5.94	**2'-MeO-PCB3**	16.61
**6'-MeO-PCB83**	2.97	**4-MeO-PCB11**	42.88
**4'-MeO-PCB93**	6.91	**4'-MeO-PCB25**	16.16
**4'-MeO-PCB101**	4.75	**4-MeO-PCB52**	40.94
**2'-MeO-PCB106 +2'-MeO-PCB 114**	3.72		

Table S3. LOQs (95% quantile of 18 method blanks) for East Chicago, IN, N=18

congener name	pg/mL of 1% KCl	congener name	pg/mL of 1% KCl
**2-MeO-PCB2**	2.7	**4'-MeO-PCB120**	25.33
**2'-MeO-PCB2**	4.1	**4'-MeO-PCB86**	11.53
**6-MeO-PCB2**	2.5	**4'-MeO-PCB97**	18.28
**4-MeO-PCB1**	10.7	**4'-MeO-PCB108**	14.00
**5-MeO-PCB2**	1.6	**3-MeO-PCB118**	9.59
**3'-MeO-PCB2**	10.7	**4-MeO-PCB107**	20.80
**4-MeO-PCB2**	17.7	**4'-MeO-PCB127**	14.88
**4'-MeO-PCB2**	29.6	**4-MeO-PCB134**	6.26
**4'-MeO-PCB3**	40.7	**4-MeO-PCB146**	6.62
**2'-MeO-PCB5**	3.6	**3'-MeO-PCB138**	5.93
**3'-MeO-PCB9**	1.7	**4'-MeO-PCB130**	3.01
**4'-MeO-PCB9+ 4-MeO-PCB14**	3.9	**4-MeO-PCB163**	5.06
		**5-MeO-PCB138**	6.98
**2'-MeO-PCB12**	15.4	**4'-MeO-PCB159**	4.09
**2'-MeO-PCB30**	5.0	**4-MeO-PCB162**	6.26
**3'-MeO-PCB30**	3.8	**3'-MeO-PCB184**	10.71
**6'-MeO-PCB26**	7.6	**4-MeO-PCB178**	4.49
**4'-MeO-PCB18**	26.7	**3'-MeO-PCB183**	7.13
**4'-MeO-PCB30**	13.0	**3'-MeO-PCB182**	6.65
**4'-MeO-PCB26**	9.5	**5-MeO-PCB183**	14.88
**3-MeO-PCB54**	5.1	**4-MeO-PCB187**	6.43
**2'-MeO-PCB65+ 6'-MeO-PCB69**	4.1	**4-MeO-PCB177**	6.98
		**3'-MeO-PCB180**	6.18
**4-MeO-PCB65**	6.1	**4'-MeO-PCB172**	5.08
**3'-MeO-PCB65**	10.8	**4'-MeO-PCB172**	8.40
**2'-MeO-PCB61**	24.9	**4-MeO-PCB202**	9.44
**4'-MeO-PCB72**	10.5	**4'-MeO-PCB201**	7.27
**4'-MeO-PCB69**	15.2	**4'-MeO-PCB198+ 4'-MeO-PCB200+ 3'-MeO-PCB203**	3.98
**4'-MeO-PCB65**	4.2		
**3'-MeO-PCB61**	8.7		
**4'-MeO-PCB61**	5.6	**4'-MeO-PCB199**	6.64
**4'-MeO-PCB79**	8.4	**4'-MeO-PCB208**	13.41
**6'-MeO-PCB101**	10.0	**2'-MeO-PCB3**	6.07
**6'-MeO-PCB83**	2.7	**4-MeO-PCB11**	9.87
**4'-MeO-PCB93**	8.1	**4'-MeO-PCB25**	19.33
**4'-MeO-PCB101**	11.4	**4-MeO-PCB52**	43.41
**2'-MeO-PCB106 + 2'-MeO-PCB114**	7.4		

Table S4. Composition of PCB sulfate congener groups

Congener Group #	PCB Sulfates Included	Congener Group#	PCB Sulfates Included
**1**	4-PCB1 Sulfate	**107**	4-PCB107 Sulfate
**2**	2-PCB2 Sulfate + 2'-PCB2 Sulfate + 6-PCB2 Sulfate + 5-PCB2 Sulfate + 3'-PCB2 Sulfate + 4-PCB2 Sulfate + 4'-PCB2 Sulfate	**108**	4'-PCB108 Sulfate
**3**	4'-PCB3 Sulfate + 2'-PCB3 Sulfate	**118**	3-PCB118 Sulfate
**5**	2'-PCB5 Sulfate	**120**	4'-PCB120 Sulfate
**9**	4'-PCB9 Sulfate+ 4-PCB 14 Sulfate + 3'-PCB9 Sulfate	**127**	4'-PCB127 Sulfate
**11**	4-PCB11 Sulfate	**130**	4'-PCB130 Sulfate
**12**	2'-PCB12 Sulfate	**134**	4-PCB134 Sulfate
**18**	4'-PCB18 Sulfate	**138**	3'-PCB138 Sulfate + 5-PCB138 Sulfate
**25**	4'-PCB25 Sulfate	**146**	4-PCB146 Sulfate
**26**	6'-PCB26 Sulfate + 4'-PCB26 Sulfate	**159**	4'-PCB159 Sulfate
**30**	2'-PCB30 Sulfate + 3'-PCB30 Sulfate + 4'-PCB30 Sulfate	**162**	4-PCB162 Sulfate
**52**	4-PCB52 Sulfate	**163**	4-PCB163 Sulfate
**54**	3-PCB54 Sulfate	**172**	4-PCB172 Sulfate + 4'-PCB172 Sulfate
**61**	2'-PCB61 Sulfate + 3'-PCB61 Sulfate + 4'-PCB61 Sulfate	**177**	4-PCB177 Sulfate
**65**	2'-PCB65 Sulfate+ 6'-PCB 69 Sulfate + 4-PCB65 Sulfate + 3'-PCB65 Sulfate + 4'-PCB65 Sulfate	**178**	4-PCB178 Sulfate
**69**	4'-PCB69 Sulfate	**180**	3'-PCB180 Sulfate
**72**	4'-PCB72 Sulfate	**182**	3'-PCB182 Sulfate
**79**	4'-PCB79 Sulfate	**183**	5-PCB183 Sulfate + 4-PCB 187 Sulfate + 3'-PCB183 Sulfate
**83**	6'-PCB83 Sulfate	**184**	3'-PCB184 Sulfate
**86**	4'-PCB86 Sulfate	**198**	4'-PCB198 Sulfate+ 4'-PCB 200 Sulfate + 3'-PCB 203 Sulfate

**93**	4'-PCB93 Sulfate	**199**	4'-PCB199 Sulfate
**97**	4'-PCB97 Sulfate	**201**	4'-PCB201 Sulfate
**101**	6'-PCB101 Sulfate + 4'-PCB101 Sulfate	**202**	4-PCB202 Sulfate
**106**	2'-PCB106 Sulfate+ 2'-PCB 114 Sulfate	**208**	4'-PCB208 Sulfate

Table S5. Frequency of detection (DF), median and range (5th-95th) of the PCB sulfates detected in mothers and children from Columbus Junction. PCB sulfates are reported as pg per g fresh weight.

		COLUMBUS JUNCTION							
		Mothers (N=12)				Children (N=12)			
**CONGENER GROUP #**	**PCB Sulfates**	**DF**	**med**	**5%**	**95%**	**DF**	**med**	**5%**	**95%**
**1**	4-PCB1 Sulfate	17%	<LOQ	<LOQ	5	17%	<LOQ	<LOQ	5
**2**	2-PCB2 Sulfate + 2'-PCB2 Sulfate + 6-PCB2 Sulfate + 5-PCB2 Sulfate + 3'-PCB2 Sulfate + 4-PCB2 Sulfate + 4'-PCB2 Sulfate	92%	187	31	795	83%	225	<LOQ	622
**3**	4'-PCB3 Sulfate + 2'-PCB3 Sulfate	33%	<LOQ	<LOQ	30	25%	<LOQ	<LOQ	55
**5**	2'-PCB5 Sulfate	25%	<LOQ	<LOQ	8	8%	<LOQ	<LOQ	2
**9**	4'-PCB9 Sulfate + 4-PCB 14 Sulfate + 3'-PCB9 Sulfate	67%	6	<LOQ	17	33%	<LOQ	<LOQ	18
**11**	4-PCB11 Sulfate	100%	583	200	3655	92%	772	43	4901
**12**	2'-PCB12 Sulfate	17%	<LOQ	<LOQ	31	8%	<LOQ	<LOQ	14
**18**	4'-PCB18 Sulfate	25%	<LOQ	<LOQ	197	17%	<LOQ	<LOQ	159
**25**	4'-PCB25 Sulfate	100%	62	17	229	100%	62	20	156
**26**	6'-PCB26 Sulfate + 4'-PCB26 Sulfate	25%	<LOQ	<LOQ	82	17%	<LOQ	<LOQ	40
**30**	2'-PCB30 Sulfate + 3'-PCB30 Sulfate + 4'-PCB30 Sulfate	17%	<LOQ	<LOQ	25	25%	<LOQ	<LOQ	4
**52**	4-PCB52 Sulfate	17%	<LOQ	<LOQ	117	8%	<LOQ	<LOQ	289

**54**	3-PCB54 Sulfate	17%	<LOQ	<LOQ	5	17%	<LOQ	<LOQ	2
**61**	2'-PCB61 Sulfate + 3'-PCB61 Sulfate + 4'-PCB61 Sulfate	58%	4	<LOQ	73	50%	1	<LOQ	34
**65**	2'-PCB65 Sulfate+ 6'-PCB 69 Sulfate + 4-PCB65 Sulfate + 3'-PCB65 Sulfate + 4'-PCB65 Sulfate	67%	3	<LOQ	25	33%	<LOQ	<LOQ	6
**69**	4'-PCB69 Sulfate	17%	<LOQ	<LOQ	10	25%	<LOQ	<LOQ	17
**72**	4'-PCB72 Sulfate	33%	<LOQ	<LOQ	13	25%	<LOQ	<LOQ	22
**79**	4'-PCB79 Sulfate	25%	<LOQ	<LOQ	11	8%	<LOQ	<LOQ	2
**83**	6'-PCB83 Sulfate	0%	<LOQ	<LOQ	0	8%	<LOQ	<LOQ	3
**86**	4'-PCB86 Sulfate	50%	4	<LOQ	15	0%	<LOQ	<LOQ	0
**93**	4'-PCB93 Sulfate	17%	<LOQ	<LOQ	12	8%	<LOQ	<LOQ	5
**97**	4'-PCB97 Sulfate	25%	<LOQ	<LOQ	12	8%	<LOQ	<LOQ	5
**101**	6'-PCB101 Sulfate + 4'-PCB101 Sulfate	58%	1	<LOQ	19	67%	7	<LOQ	39
**106**	2'-PCB106 Sulfate+ 2'-PCB 114 Sulfate	0%	<LOQ	<LOQ	<LOQ	17%	<LOQ	<LOQ	4
**107**	4-PCB107 Sulfate	33%	<LOQ	<LOQ	12	17%	<LOQ	<LOQ	18
**108**	4'-PCB108 Sulfate	42%	<LOQ	<LOQ	11	42%	<LOQ	<LOQ	5
**118**	3-PCB118 Sulfate	25%	<LOQ	<LOQ	34	17%	<LOQ	<LOQ	23
**120**	4'-PCB120 Sulfate	0%	<LOQ	<LOQ	<LOQ	17%	<LOQ	<LOQ	13
**127**	4'-PCB127 Sulfate	8%	<LOQ	<LOQ	3	0%	<LOQ	<LOQ	0
**130**	4'-PCB130 Sulfate	17%	<LOQ	<LOQ	21	8%	<LOQ	<LOQ	4
**134**	4-PCB134 Sulfate	25%	<LOQ	<LOQ	13	8%	<LOQ	<LOQ	7
**138**	3'-PCB138 Sulfate + 5-PCB138 Sulfate	17%	<LOQ	<LOQ	39	17%	<LOQ	<LOQ	37
**146**	4-PCB146 Sulfate	17%	<LOQ	<LOQ	7	8%	<LOQ	<LOQ	3

**159**	4'-PCB159 Sulfate	8%	<LOQ	<LOQ	2	17%	<LOQ	<LOQ	10
**162**	4-PCB162 Sulfate	17%	<LOQ	<LOQ	30	8%	<LOQ	<LOQ	8
**163**	4-PCB163 Sulfate	25%	<LOQ	<LOQ	11	8%	<LOQ	<LOQ	2
**172**	4-PCB172 Sulfate + 4'-PCB172 Sulfate	25%	<LOQ	<LOQ	15	25%	<LOQ	<LOQ	34
**177**	4-PCB177 Sulfate	8%	<LOQ	<LOQ	6	25%	<LOQ	<LOQ	14
**178**	4-PCB178 Sulfate	17%	<LOQ	<LOQ	4	8%	<LOQ	<LOQ	4
**180**	3'-PCB180 Sulfate	0%	<LOQ	<LOQ	<LOQ	25%	<LOQ	<LOQ	13
**182**	3'-PCB182 Sulfate	17%	<LOQ	<LOQ	53	8%	<LOQ	<LOQ	28
**183**	5-PCB183 Sulfate+ 4-PCB 187 Sulfate + 3'-PCB183 Sulfate	17%	<LOQ	<LOQ	21	17%	<LOQ	<LOQ	12
**184**	3'-PCB184 Sulfate	8%	<LOQ	<LOQ	5	8%	<LOQ	<LOQ	31
**198**	4'-PCB198 Sulfate+ 4'-PCB 200 Sulfate+ 3'-PCB 203 Sulfate	17%	<LOQ	<LOQ	6	17%	<LOQ	<LOQ	4
**199**	4'-PCB199 Sulfate	17%	<LOQ	<LOQ	21	8%	<LOQ	<LOQ	6
**201**	4'-PCB201 Sulfate	25%	<LOQ	<LOQ	11	0%	<LOQ	<LOQ	<LOQ
**202**	4-PCB202 Sulfate	8%	<LOQ	<LOQ	12	0%	<LOQ	<LOQ	<LOQ
**208**	4'-PCB208 Sulfate	8%	<LOQ	<LOQ	1	0%	<LOQ	<LOQ	<LOQ

Congeners in red: originally co-elute as MeO-PCBs

Table S6. Frequency of detection (DF), median and range (5th-95th) of the PCB sulfates detected in mothers and children from East Chicago. PCB sulfates are reported as pg per g fresh weight.

		EAST CHICAGO							
		Mothers (N=12)				Children (N=12)			
**CONGENER GROUP #**	**PCB Sulfates**	**DF**	**med**	**5%**	**95%**	**DF**	**med**	**5%**	**95%**
**1**	4-PCB1 Sulfate	25%	<LOQ	<LOQ	50	33%	<LOQ	<LOQ	109
**2**	2-PCB2 Sulfate + 2'-PCB2 Sulfate + 6-PCB2 Sulfate + 5-PCB2 Sulfate + 3'-PCB2 Sulfate + 4-PCB2 Sulfate + 4'-PCB2 Sulfate	100%	1574	550	4884	100%	982	227	4286
**3**	4'-PCB3 Sulfate + 2'-PCB3 Sulfate	83%	87	<LOQ	639	75%	38	<LOQ	860
**5**	2'-PCB5 Sulfate	17%	<LOQ	<LOQ	15	33%	<LOQ	<LOQ	16
**9**	4'-PCB9 Sulfate+ 4-PCB 14 Sulfate + 3'-PCB9 Sulfate	25%	<LOQ	<LOQ	18	25%	<LOQ	<LOQ	10
**11**	4-PCB11 Sulfate	100%	345	77	2322	92%	370	14	1717
**12**	2'-PCB12 Sulfate	8%	<LOQ	<LOQ	<LOQ	0%	<LOQ	<LOQ	<LOQ
**18**	4'-PCB18 Sulfate	58%	23	<LOQ	155	50%	<LOQ	<LOQ	272
**25**	4'-PCB25 Sulfate	83%	139	<LOQ	297	92%	78	8	243
**26**	6'-PCB26 Sulfate + 4'-PCB26 Sulfate	25%	<LOQ	<LOQ	48	25%	<LOQ	<LOQ	62
**30**	2'-PCB30 Sulfate + 3'-PCB30 Sulfate + 4'-PCB30 Sulfate	42%	<LOQ	<LOQ	37	33%	<LOQ	<LOQ	59
**52**	4-PCB52 Sulfate	75%	43	<LOQ	301	83%	114	<LOQ	399
**54**	3-PCB54 Sulfate	8%	<LOQ	<LOQ	3	17%	<LOQ	<LOQ	11

**61**	2'-PCB61 Sulfate + 3'-PCB61 Sulfate + 4'-PCB61 Sulfate	58%	6	<LOQ	47	50%	1	<LOQ	18
**65**	2'-PCB65 Sulfate+ 6'-PCB 69 Sulfate + 4-PCB65 Sulfate + 3'-PCB65 Sulfate + 4'-PCB65 Sulfate	42%	<LOQ	<LOQ	31	58%	2	<LOQ	23
**69**	4'-PCB69 Sulfate	25%	<LOQ	<LOQ	33	25%	<LOQ	<LOQ	33
**72**	4'-PCB72 Sulfate	8%	<LOQ	<LOQ	0	0%	<LOQ	<LOQ	0
**79**	4'-PCB79 Sulfate	8%	<LOQ	<LOQ	10	0%	<LOQ	<LOQ	0
**83**	6'-PCB83 Sulfate	0%	<LOQ	<LOQ	0	17%	<LOQ	<LOQ	5
**86**	4'-PCB86 Sulfate	17%	<LOQ	<LOQ	39	0%	<LOQ	<LOQ	0
**93**	4'-PCB93 Sulfate	25%	<LOQ	<LOQ	13	33%	<LOQ	<LOQ	19
**97**	4'-PCB97 Sulfate	17%	<LOQ	<LOQ	28	17%	<LOQ	<LOQ	17
**101**	6'-PCB101 Sulfate + 4'-PCB101 Sulfate	67%	19	<LOQ	66	33%	<LOQ	<LOQ	21
**106**	2'-PCB106 Sulfate+ 2'-PCB 114 Sulfate	17%	<LOQ	<LOQ	12	25%	<LOQ	<LOQ	20
**107**	4-PCB107 Sulfate	25%	<LOQ	<LOQ	48	17%	<LOQ	<LOQ	70
**108**	4'-PCB108 Sulfate	25%	<LOQ	<LOQ	62	17%	<LOQ	<LOQ	72
**118**	3-PCB118 Sulfate	17%	<LOQ	<LOQ	15	8%	<LOQ	<LOQ	7
**120**	4'-PCB120 Sulfate	33%	<LOQ	<LOQ	89	33%	<LOQ	<LOQ	73
**127**	4'-PCB127 Sulfate	8%	<LOQ	<LOQ	0	8%	<LOQ	<LOQ	10
**130**	4'-PCB130 Sulfate	42%	<LOQ	<LOQ	11	25%	<LOQ	<LOQ	9
**134**	4-PCB134 Sulfate	33%	<LOQ	<LOQ	17	42%	<LOQ	<LOQ	52
**138**	3'-PCB138 Sulfate + 5-PCB138 Sulfate	50%	1	<LOQ	16	58%	13	<LOQ	25
**146**	4-PCB146 Sulfate	0%	<LOQ	<LOQ	0	8%	<LOQ	<LOQ	8
**159**	4'-PCB159 Sulfate	25%	<LOQ	<LOQ	13	42%	<LOQ	<LOQ	18
**162**	4-PCB162 Sulfate	8%	<LOQ	<LOQ	5	17%	<LOQ	<LOQ	13

**163**	4-PCB163 Sulfate	17%	<LOQ	<LOQ	7	67%	9	<LOQ	23
**172**	4-PCB172 Sulfate + 4'-PCB172 Sulfate	25%	<LOQ	<LOQ	30	33%	<LOQ	<LOQ	30
**177**	4-PCB177 Sulfate	33%	<LOQ	<LOQ	24	25%	<LOQ	<LOQ	21
**178**	4-PCB178 Sulfate	33%	<LOQ	<LOQ	36	50%	3	<LOQ	14
**180**	3'-PCB180 Sulfate	25%	<LOQ	<LOQ	23	8%	<LOQ	<LOQ	0
**182**	3'-PCB182 Sulfate	17%	<LOQ	<LOQ	37	25%	<LOQ	<LOQ	7
**183**	5-PCB183 Sulfate+ 4-PCB 187 Sulfate + 3'-PCB183 Sulfate	33%	<LOQ	<LOQ	39	33%	<LOQ	<LOQ	64
**184**	3'-PCB184 Sulfate	17%	<LOQ	<LOQ	8	25%	<LOQ	<LOQ	28
**198**	4'-PCB198 Sulfate+ 4'-PCB 200 Sulfate+ 3'-PCB 203 Sulfate	8%	<LOQ	<LOQ	1	0%	<LOQ	<LOQ	<LOQ
**199**	4'-PCB199 Sulfate	25%	<LOQ	<LOQ	29	8%	<LOQ	<LOQ	5
**201**	4'-PCB201 Sulfate	0%	<LOQ	<LOQ	<LOQ	8%	<LOQ	<LOQ	7
**202**	4-PCB202 Sulfate	0%	<LOQ	<LOQ	<LOQ	17%	<LOQ	<LOQ	36
**208**	4'-PCB208 Sulfate	17%	<LOQ	<LOQ	10	8%	<LOQ	<LOQ	15

Congeners in red: originally co-eluted as MeO-PCBs

Table S7. Correlation analysis of PCB sulfate concentrations in mothers-children dyads. Analysis was determined from 12 mother-child dyads (6 mother-daughter and 6 mother-son) from each of the two populations. Numbers are correlation coefficients (p values). P-values were obtained from simple regression analysis. Significant correlation coefficients (i.e., $p<0.05$) are in bold. Total PCB 2 Sulfates include 2-PCB 2 Sulfate + 2'-PCB 2 Sulfate + 6-PCB 2 Sulfate + 5-PCB 2 Sulfate + 3'-PCB 2 Sulfate + 4-PCB 2 Sulfate + 4'-PCB 2 Sulfate.

	**Columbus Junction, IA**	**East Chicago, IN**
Total PCB Sulfates	0.21 (0.50)	0.49 (0.11)
Total PCB 2 Sulfates	**0.83 (0.0009)**	0.12 (0.70)
4-PCB 2 Sulfate	**0.85 (0.0005)**	0.12 (0.72)
4'-PCB 2 Sulfate	0.56 (0.059)	0.28 (0.39)
2'-PCB 3 Sulfate	0.17 (0.60	**0.70 (0.011)**
4'-PCB 3 Sulfate	0.11 (0.73)	0.039 (0.90)
4-PCB 11 Sulfate	0.35 (0.27)	**0.89 (0.0001)**
4'-PCB 18 Sulfate	**0.78 (0.003)**	**0.83 (0.0009)**
4'-PCB 25 Sulfate	0.012 (0.97)	0.28 (0.39)
4-PCB 52 Sulfate	0.44 (0.16)	0.23 (0.46)

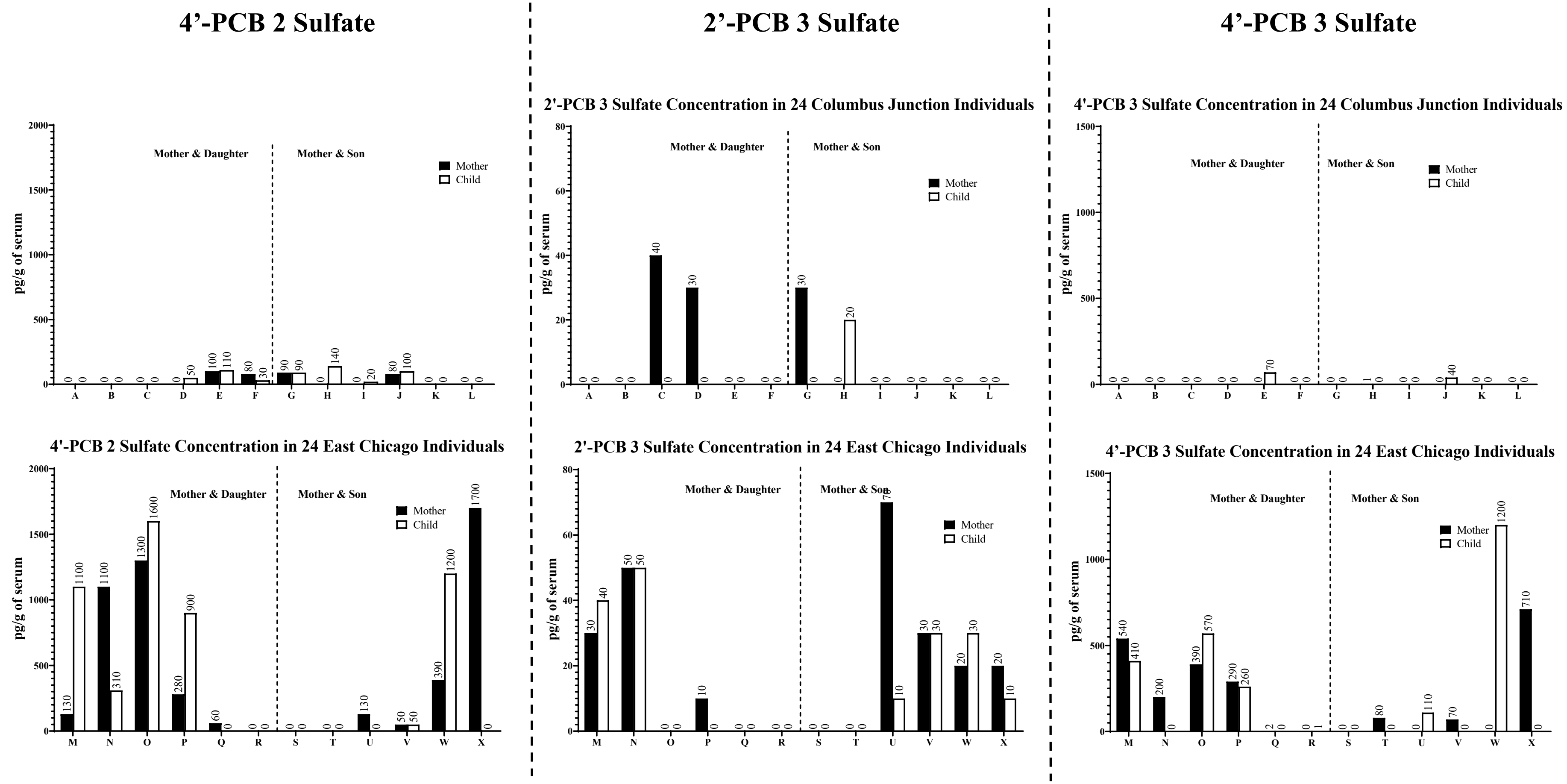

Figure S1. Comparisons of 4’-PCB 2 sulfate, 2’-PCB 3 sulfate, and 4’-PCB 3 sulfate concentrations in 48 individuals from the Columbus Junction and East Chicago cohorts. Statistical p values between the two cohorts for each of these three compounds are 0.002 for 4’-PCB 2 sulfate, 0.01 for 2’-PCB 3 sulfate, and 0.003 for 4’-PCB 3 sulfate.

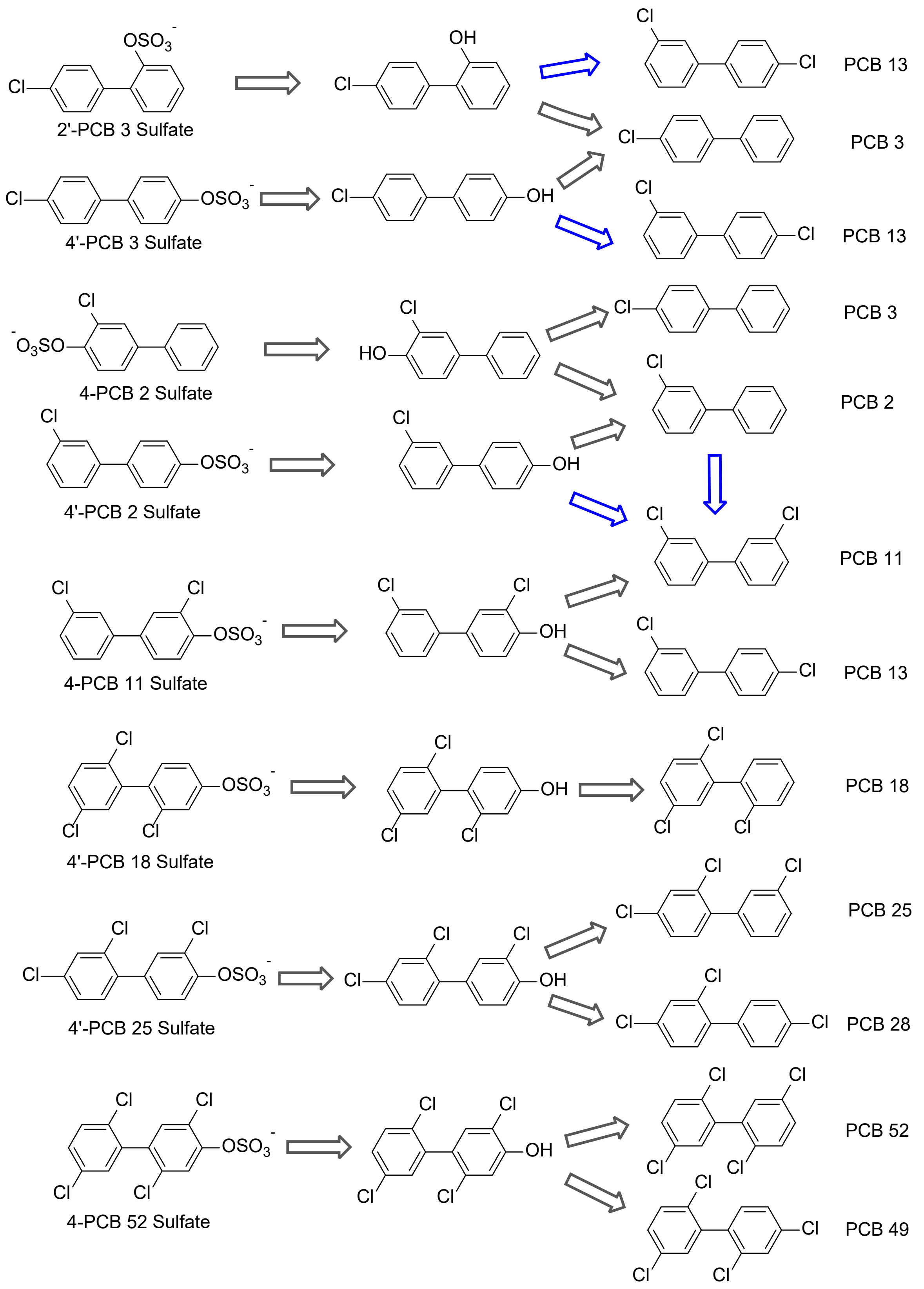

Figure S2. Preliminary proposed retro-analysis of 8 major PCB sulfates to their parent PCBs. Blue arrows indicate a dechlorination reaction involved. Note: Dechlorination (via mammalian or intestinal microflora reactions), oxidation, and sulfation following exposure to PCBs 92, 95, and/or 101 might also be potential additional sources of 4-PCB 52 sulfate.

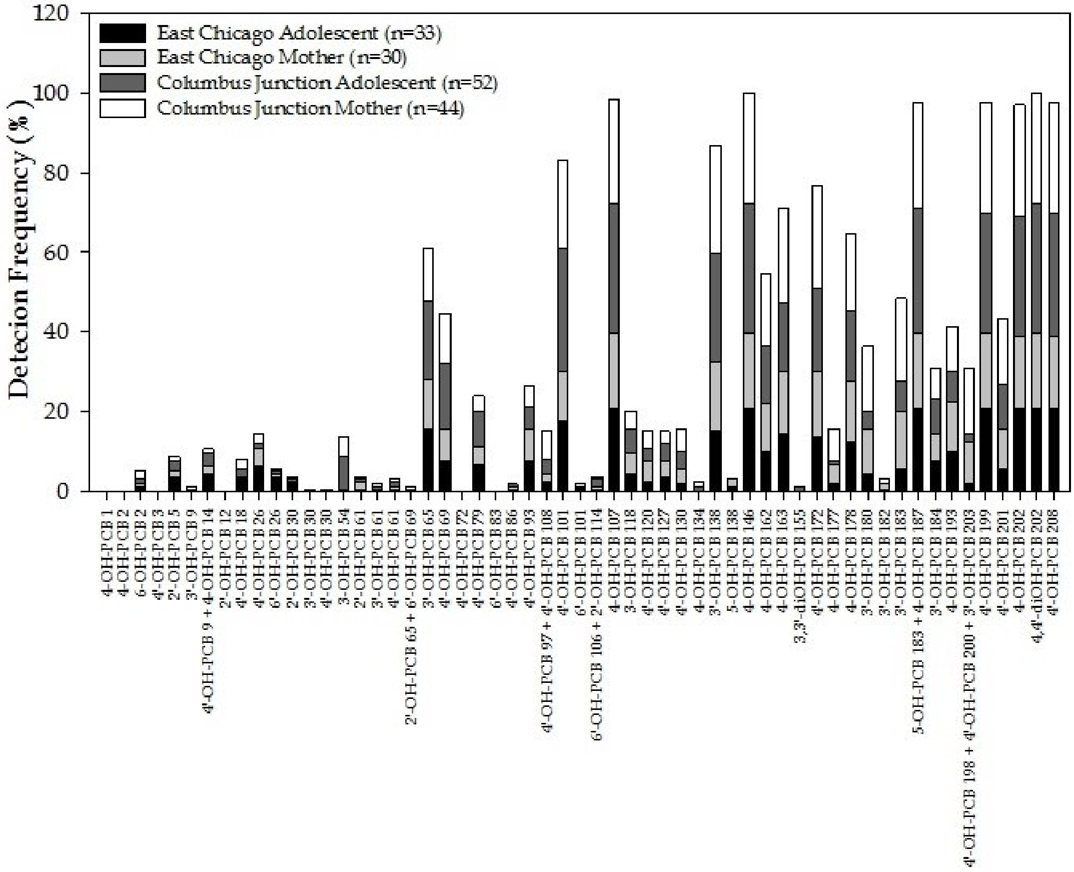

Figure S3. Detection frequency of OH-PCB congeners in East Chicago and Columbus Junction adolescents and their mothers. (Koh, W.X., *et al. Chemosphere* **2016**, *147*, 389-395)

Reprinted with permission from: Koh, W. X.; Hornbuckle, K. C.; Marek, R. F.; Wang, K.; Thorne, P. S., Hydroxylated polychlorinated biphenyls in human sera from adolescents and their mothers living in two US Midwestern communities. *Chemosphere* **2016,** *147*, 389-395. [Automatic permission from Elsevier as an STM signatory publisher]
